# Preventative therapy for breast cancer: a clinical experience

**DOI:** 10.1007/s10549-023-06985-1

**Published:** 2023-06-19

**Authors:** Rebekah Law, Katherine Krupa, Jennifer Rusby

**Affiliations:** 1 The Royal Marsden Hospital, London, UK; 2 Institute of Cancer Research, London, UK

**Keywords:** Breast cancer prevention, Chemoprophylaxis, Chemoprevention, Preventative therapy

## Abstract

**Background:**

Breast Cancer incidence in the UK is estimated to rise to 71,000 per year by 2035. Preventative strategies could significantly reduce this. Preventative therapy reduces women’s risk of oestrogen receptor positive breast cancer, but uptake remains low. Having established a preventative therapy clinic as part of a wider breast cancer prevention project, we explored qualitative data to inform future preventative efforts.

**Method:**

Women aged 30 to 60 who had benign diagnoses at a symptomatic breast clinic or were under mammographic surveillance in the moderate risk family history clinic were invited to participate in the study. Those who expressed an interest and completed an initial questionnaire had their breast cancer risk calculated using the IBIS risk calculator. Those at increased risk were invited to a consultation about preventative therapy.

**Results:**

182 women were identified as increased risk (≥ 17% lifetime or ≥ 3% 10-year risk NICE guidelines: Familial breast cancer: classification, care and managing breast cancer and related risks in people with a family history of breast cancer, 2013^1^) of whom 91 women (50%) would not have been identified by family history criteria alone. 96% attended a risk/prevention consultation and all eligible women accepted screening mammography but only 14 (8%) women requested a preventative therapy prescription during the duration of the study. Reluctance to take medication and inconvenient time of life were common reasons for declining preventative therapy. Despite this, the majority were grateful for breast cancer risk and prevention information.

**Conclusions:**

Women at increased risk of breast cancer accept additional screening but are reluctant to take preventative therapy. This suggests that stratified screening methods using risk calculations would have high uptake. Raising awareness of preventative therapy is important and the breast cancer community has yet to find the optimum timing and formula for discussing it and must accept women’s informed preferences above artificial targets.

**Registration numbers:**

The PIONEER study was granted Health Research Authority (HRA) ethical approval by the Westminster Ethics Committee. IRAS project ID 265619, ClinicalTrials.gov Identifier: NCT04574063. Recruitment began in September 2020 and was completed in October 2021.

## Introduction

Breast Cancer is common: Data from 2015 to 2017 suggest a UK incidence of 55,200 per year [2]. It is estimated that by 2035 the incidence could exceed 70,000 per year [3]. Such a high incidence suggests that even small percentage reductions achieved by preventative strategies could have a significant impact on breast cancer incidence. It is estimated that 23% of breast cancers could be prevented through lifestyle change [4] and further breast cancers could be prevented through the use of preventative therapy in women who are at increased risk breast cancer [5]. However, uptake of preventative therapy has not become mainstream despite the FDA first approving it in 1998 [6] and National Institute for Health and Clinical Excellence (NICE) recommending it in 2013.

In 2013 a meta-analysis of nine trials (Marsden, IBIS-1, NSABP-P-1, Italian, CORE/MORE, RUTH, STAR, PEARL, GENERATIONS) was performed to assess the effectiveness of selective oestrogen receptor modulators (SERMs) in prevention of breast cancer. This indicated that women at increased risk of developing breast cancer who were given SERMs had a 30% reduction in relative risk [7]. In the post-menopausal setting, aromatase inhibitors could reduce breast cancer risk by 50% [7, 8]. Importantly, none of these studies demonstrated a reduction in mortality [6]. Freedman et al. suggested that 15.5% of women aged 35–79 in the US in 2000 could be eligible for preventative therapy [9], but in 2016 only 0.03% of American women were taking tamoxifen, and 0.21% were taking raloxifene [10].

A crude calculation of the potential population impact of preventative therapy for breast cancer in the UK suggests that if 15.5% of women were eligible and all took tamoxifen, more than 250,000 breast cancers could be prevented over the course of these women's lives. As 17% is the threshold for being considered at increased risk, many women at increased risk would have a higher lifetime risk than this and stand to benefit more. More than 250,000 could therefore be considered a conservative estimate. While statistics from the US may not be directly applicable to the UK population, these figures do give an indication of the number of women who potentially could be spared a cancer diagnosis and all the anxiety, treatment and side effects that this invokes.

In the UK, NICE introduced guidance in 2013 advising clinicians to offer preventative therapy to women at increased risk of breast cancer. Hackett et al. [11] performed a prospective multicentre study and found that uptake of preventative therapy was 14.7%. The same paper reported that family priorities played a big part in women’s decisions about whether to take preventative therapy. [11]

Between 2015 and 2020, part of the Cancer Strategy for England was to recommend that general practitioners (GPs) prescribe tamoxifen for breast cancer primary prevention, and yet only half of GPs surveyed knew that tamoxifen could be used for preventative therapy. [12] Smith et al. concluded that giving the first tamoxifen prescription in secondary care may overcome some barriers in prescribing. [12]

**Fig. 1:**
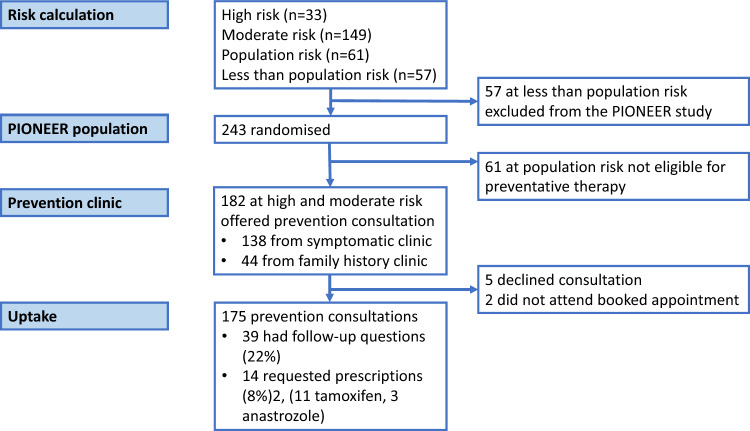
Consort diagram of recruitment to the PIONEER lifestyle breast cancer prevention pilot study and subsequent consultations for women at increased risk

**Fig. 2:**
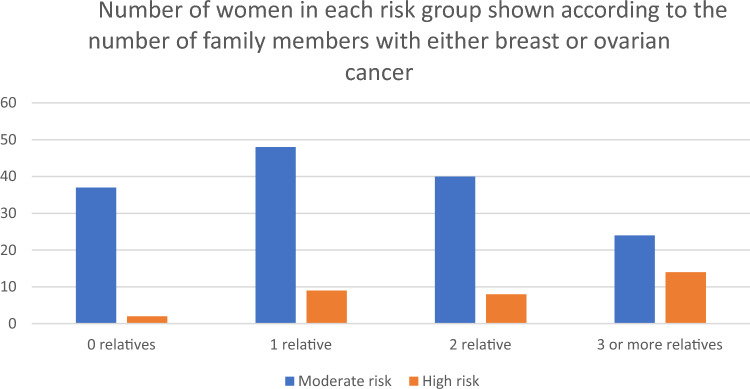
Percentage of women in each risk group compared to number of family members with either breast cancer or ovarian cancer

The UK Academic Health Science Network (AHSN) announced in March 2021 that tamoxifen for preventative therapy would become an NHS England “Rapid Uptake Product” [13] because it has approval yet its use had not become widespread. Although this plan was subsequently reversed, this suggests that, while the conversation about preventative therapy is not new, it is still very topical. As part of a randomised controlled pilot study to encourage lifestyle change to reduce breast cancer risk, we calculated participants’ risk of developing breast cancer using the Tyrer–Cuzick (IBIS) model [14]. NICE guidance states that all those at increased risk of developing breast cancer should be offered a conversation about chemoprevention [1] and we therefore had a duty of care to offer this to women identified through this study. A preventative therapy clinic was established to address this need. During these conversations, we identified several themes which prevent women from starting preventative therapy. Dissemination of these themes may be beneficial to future preventative efforts (Figs. 1 and 2).

## Methods

Women aged 30–60 who were assessed in the symptomatic breast clinic and discharged with a benign or normal diagnosis were invited to join the PIONEER study (ClinicalTrials.gov Identifier: NCT04574063). This was a randomised controlled pilot study which invited women to engage in lifestyle change to reduce breast cancer risk. Benign diagnoses were defined as anything other than cancer or DCIS. Women under surveillance in our moderate risk family history clinic were also invited to participate. They were given a participant information leaflet. The onus was on the participant to contact the study team should they wish to take part.

Once contact had been made, eligibility was confirmed (see criteria below) and a link was sent by email to an online consent form and initial questionnaire. The participant was given a unique ID to complete the questionnaire. The questionnaire covered lifestyle risk factors and included questions needed to complete the Tyrer–Cuzick (IBIS) breast cancer risk calculation (Version 8b), which was the calculator used in the PIONEER protocol. Mammographic density was also included for women who were over the age of 40 and had a mammogram as part of their clinical assessment.

Those found to be at increased risk of developing breast cancer using the Tyrer–Cuzick risk calculator (moderate risk ≥ 3% 10-year risk or ≥ 17% lifetime risk *or* high-risk ≥ 8% ten-year risk or ≥ 30% lifetime risk) [1] were invited to a consultation about preventative therapy. As the NICE guidance does not specify whether to preferentially use 10-year or lifetime risk as an indication for preventative therapy, the highest risk category was counted. Of note, the hospital was permitted to bill for these clinics as they meet NICE guidance.

Box 1: Inclusion criteria for PIONEER study Eligibility criteria for the PIONEER study.

- Female
- Benign or normal diagnosis
- Aged 30–60
- Willing to take part in a year-long lifestyle intervention
- No previous or current malignancy

## Preventative therapy appointments

During the COVID pandemic these clinic appointments were performed by telephone. Consultations were based on the structure shown in box 2. After the consultation, women were sent the appropriate NICE decision aid [16] about preventative therapy and the hospital’s own ‘chemoprevention’ information leaflet.

The main researcher, RL, carried out all these consultations and kept notes of comments and themes that arose during these conversations. Formal recording, transcription and qualitative analysis were not performed as this was not the main focus of this study, but the themes are presented here to inform plans for future prevention programs.

Box 2: Typical structure of a preventative therapy consultation [15, 16]

- Briefly introduce the concepts of 10-year and lifetime risk
- Communicate the patient’s own risk result and compare this to general population risk.
- Explain which factors may have put the patient at increased risk.
- Explain that preventative therapy has been shown to reduce relative risk by 30%-50% depending on agent[16].
- Explain risks and side effects of medication.
  - If women are particularly worried about side effects, explain that not all women experience side effects, and the drug can be stopped if side effects are troublesome.
  - If women have dense breast tissue explain that tamoxifen reduces breast density [15] which would make detection easier.

## Results

243 women were recruited to the main study via the diagnostic clinic and family history clinics. Of these, 149 (61%) were found to be moderate risk and 33 (14%) were found to be high-risk. All were offered a telephone consultation. Five women declined the consultation and two women did not attend their appointment (Fig.1). 

### Family history

Of women identified as having increased risk of breast cancer using the Tyrer–Cuzick (IBIS) calculator, only 91 (50%) would have been identified by family history alone.

Women at high-risk (rather than moderate risk) tended to have a stronger family history of breast or ovarian cancer.(Fig.2) Twenty-two (66%) of the high-risk women had at least two family members who had breast or ovarian cancer. Sixty-four (42%) moderate risk women had at least two family members with a history of breast or ovarian cancer. Thirty-seven (25%) moderate risk women had no family history. Their risks were increased by other risk factors in the IBIS risk calculator, such as previous biopsy or mammographic density.

### Ten year and lifetime risk

Using the Tyrer–Cuzick Model, it was calculated that 63 (42%) women were at moderate 10-year risk of breast cancer but had a lifetime risk similar to that of the general population. The mean age of these women was 53.

Using the Tyrer–Cuzick Model, it was calculated that 13 women were at moderate lifetime risk of breast cancer, but their 10-year risk was similar to that of the general population. The mean age of these women was 35.

If participants had either increased lifetime or increased 10-year risk, they were eligible for preventative therapy.

### Breast density

Most women in the high-risk groups had dense breast tissue (BIRADS C or D). Twenty-seven women had not had a mammogram as they were under the age of 40.

### Uptake

Of the 182 women at high or moderate risk, 175 women accepted the offer of a preventative therapy consultation. Five declined and 2 did not attend an appointment. 11of 118 women up to the age of 50 (9%) have requested tamoxifen prescriptions and 3 over 50s (5%) women have said they would like to take anastrozole. Two women are at ‘high-risk’ and 12 women are at ‘moderate-risk’. The mean age of uptake was 47.4 with a range of 40–55. Of note, requests for prescriptions were not always immediate, with one woman taking nearly a year to request a prescription.

The offer of enhanced mammographic surveillance was accepted by all women aged between 40 and 50, who had not already been offered this through the family history clinic (*N* = 37).

### Themes

#### Gratitude for the information

This theme is embodied in a quote: “Thank you so much for the information. I think what you’re doing is brilliant. It’s given me a lot to think about”. Another woman stated: “What a brilliant service to have a one-to-one conversation. I could read all you’ve said in a newspaper, but it wouldn’t feel so individual to me.”

Almost all women expressed gratitude for the information provided and for the sense of certainty that a percentage risk gave them. This was despite an extensive initial explanation that we cannot be certain that any woman’s risk is exactly that of the calculation. One woman felt that her risk score validated what she felt she already knew which in turn gave a sense of reassurance. These participants’ agenda seemed more to do with managing anxiety than managing risk.

#### “I didn’t know medication could reduce [breast cancer] risk”

None of the women recalled having heard of preventative therapy as a concept. This highlights a lack of awareness in the general population around preventative therapy. Indeed, many women were surprised when preventative therapy was mentioned and said things like: ‘I thought you were going to tell me to lose weight or drink less.’

#### “I don’t like taking medicines”

One theme that has emerged strongly is that women ‘don’t like taking medicine’. One woman reported that she would rather “suffer through a headache than take a paracetamol”. Another stated that she would “prefer to use healthy living and ‘natural’ methods to improve [her] health”. Before I was able to speak about preventative therapy another woman reported that she was unwilling to take ‘experimental’ medicine.

#### “I pay for my prescriptions. Would I have to pay for this?”

One person highlighted the cost of prescriptions as a potential barrier. In England, most patients will pay for prescription medications. The current cost for a prescription is £9.35. Prescriptions are often made monthly, to allow for reviews of medication. If a woman were to pay £9.35 each month for 5 years, the cost of this medication would be £561. For some this may be a trivial amount of money, but for others, this may be the deciding factor in whether to start taking preventative therapy.

#### “It’s not the right time for me”

Another theme that emerged was one of deferring a decision about preventative therapy. A number of women reported either that they were trying to have a baby or were unsure whether they had completed their family. One woman reported that she was struggling with her mental health and didn’t feel able to ‘rock the boat’ by adding a new medication into the mix. Other women were struggling with menopausal symptoms and wanted to see if these would settle down before considering preventative therapy. Two women wanted to discuss the possibility of starting preventative therapy with their partner. One woman stated that she had children with special needs. She stated, “right now, I have to be as well as possible for my children.” She went on to say that her day-to-day life was very demanding in this stage of life. One woman waited 11 months to contact the team asking for a prescription, suggesting that some women want to take time to make this decision.

#### Deferring the decision in preference for anastrozole

Some women who were perimenopausal, asked if they could still take tamoxifen after the menopause. This led to discussions around anastrozole. Anastrozole reduces the risk of breast cancer by 50% if taken daily for 5 years. This additional risk reduction seemed to give pause for thought. “Is it worth waiting till I’m through the menopause so that I can get extra risk reduction?”.

#### Fatalism

The participant with the highest risk calculated stated “I’ll probably get breast cancer one day”, but she remained unconvinced about preventative therapy. Some women said words to the effect of ‘what will be will be’.

#### Concern about implications for future treatment

Three women, who had had close contact with someone with breast cancer, asked whether taking tamoxifen would reduce their treatment options should they go onto develop breast cancer anyway. Explaining the difference between hormone positive and hormone negative cancers and the possibility that “preventative” therapy may in fact be treatment of pre-clinical disease, can be confusing. The mechanisms of action of preventative therapies are not fully understood, and so many women’s questions are answered with ‘we don’t know’. This uncertainty around some aspects of preventative therapy may be off-putting for some.

#### Side effects and risks

Women who had seen those close to them suffer with side effects of tamoxifen often cited this as a concern, though rarely was this presented as the only reason for not taking preventative therapy. Two women were unable to take tamoxifen due to a history of a clotting disorder. One woman stated, “it’s a shame that [tamoxifen] doesn’t reduce menopausal symptoms—then it would be a no brainer!”.

One woman stated that she was concerned by the increased risk of endometrial cancer. Statistics from the NICE decision aid were quoted, which state endometrial cancer risk rises from 0.3% to 0.6%. This was of greater concern than the risk of DVT and was also of greater concern than her existing moderate breast cancer risk. Another woman was concerned about her history of endometriosis and the risk, if any, that this posed on her endometrial cancer risk.

#### “Can I take HRT?”

“I know I’m at increased risk of breast cancer, but I’m really struggling with my menopausal symptoms. Can I take HRT?” This is a question that was surprising given the context of the conversation. It highlights that breast cancer risk is not at the forefront of many women’s minds, even if they are at increased risk of developing breast cancer. The future risk of breast cancer is eclipsed by difficulties with day-to-day life now.

#### False reassurance through risk calculation

One woman whose lifetime breast cancer risk was over 30%, said that she had thought her risk was more like 60%, so was reassured by the conversation. Despite this “reassurance”, at the end of the conversation she said, “sign me up! I’m not afraid of things that are going to make me better off”. Interestingly this same participant had recently improved her lifestyle by increasing her exercise and improving her diet. However, there is a possibility that women who overestimate their risk may feel that their lifestyle is validated by receiving a lower calculated risk than they anticipate. Fortunately, this was not the case for this participant.

#### “My risk isn’t that much higher than the rest of the population”

Some women who only had a slight increase in either their 10-year or lifetime risk, were comfortable that their risk was only slightly above the rest of the population and did not think their breast cancer risk outweighed the downsides of preventative therapy (Table 1).

**Table 1:** Baseline data for moderate and high-risk participants of the PIONEER study

Characteristic		Number (%)
Age range	30-40 41-50 51-60	29 (16) 89 (49) 64 (35)
Ethnicity	Arabic Asian / British Asian Black / African / Caribbean / Black British Mixed White Other	2 (1) 10 (6) 2 (1) 5 (3) 162 (89) 1 (1)
Risk group	Moderate High	149 (82) 33 (18)
Previous biopsy	Yes No	58 (32) 124 (68)
BIRADs score	A B C D No data	8 (4) 45 (25) 63 (35) 9 (21) 27 (15)
Recruitment source	Symptomatic clinic Family history	138 (76) 44 (24)

### Opportunity for further discussion

Although only one formal appointment was offered, women were invited to ask additional questions and 39 women contacted us by email following their preventative therapy conversation. Their questions covered issues such as interaction with family planning arrangements. The need for repeated discussion and time to consider the information given suggests that provision of information before the clinic appointment may improve uptake at the time of the consultation.

## Discussion

This work has demonstrated that the uptake of preventative therapy among women at increased risk of breast cancer in a dedicated clinic is low and reasons for this are diverse, some amenable to improvement, and others not. Given the estimated number of women eligible in the general population, it is likely that lack of awareness is a major factor despite this option being widely reported in mainstream press over the last decade [17–19].This can be addressed in primary and secondary care as well as by breast cancer charities.

Strengths of this study include the large number of consultations conducted by a specialist researcher, and the open lines of communication allowing follow-up questions and subsequent requests for prescription. However, there are limitations to this work. Firstly, this preventative therapy clinic was created to fulfil a duty of care to women who were found to be at increased risk while participating in a pilot of lifestyle change to reduce breast cancer risk. As such, no formal qualitative methods were used to collect data. Nonetheless, interesting themes have emerged which may be of use in designing similar clinics in the future. Furthermore, women who had a consultation had already volunteered themselves to take part in a lifestyle change study and may represent a particularly motivated group. Conversely, given the primary aim of the study was lifestyle change, women may have been reluctant to deviate from lifestyle modification alone and take medication. Either way, these factors may limit the generalisability of the results. The majority of participants were white and, while this is representative of the local population, a more ethnically diverse population may result in different findings.

While it was estimated by Freedman et al.[9] that 15.5% of women are at increased risk of breast cancer, when we take into account all women who had their risk calculated, 60% of women who consented to take part in the PIONEER study were at increased risk. There are several possible explanations of this. First, the Tyrer–Cuzick model has been reported to over-estimate risk [20]. Second, the 15.5% estimate dating from the year 2000 may now be an underestimate given that obesity has become more prevalent over time [21]. Finally, we believe that women with a family history or other breast cancer risk factors self-selected into this breast cancer risk reduction study. While uptake of chemoprevention was low in our prevention clinic, this self-selection could be harnessed in the future if symptomatic clinics included routine risk assessment using automated processes. Those who are interested in discussions about preventative therapy could self-refer for further discussion.

While few women took up the offer of a medication to reduce the risk of breast cancer, all women were keen to accept additional mammographic screening which would marginally increase their risk of breast cancer but would give them “peace of mind”. This paradox was emphasised to women in the study. Women’s eagerness for screening suggests that identification of women at moderate and high-risk could lead to substantial uptake of more frequent mammography among such women. It remains to be seen whether this would translate to improved breast cancer survival.

All but five of the eligible women were open to discussion suggesting an appetite to hear about risk reduction options. Before recruitment began, the study team had been optimistic that a designated preventative therapy clinic would lead to a greater uptake of preventative therapy than hurried symptomatic or family history clinics had yielded to date, because of the dedicated 30-min discussion with a clinician, who has risk communication training. However, tamoxifen and anastrozole uptake was only 9% and 6% in the relevant age groups. By comparison 14.7% tamoxifen uptake was found by Hackett et al.[11] 67% of Hackett’s study population were from a family history clinic compared with only 24% in our study. It is possible that family history clinics provide ‘priming’ regarding breast cancer risk and preventative therapy. Another difference is that Hackett et al. report that participant risk was “provided by clinic staff”, but the calculator used is not reported. It is possible that an overestimation in our cohort by the Tyrer–Cuzick model [20] contributed to a reduced uptake compared with Hackett et al. As with our clinic, Hackett et al. placed no age limits on starting chemoprevention. Nonetheless, in both studies, childbearing was a major factor in uptake.

A retrospective study of high-risk women in the USA showed 24% had a current or past history of chemoprevention use [22]. Of note, high-risk was defined based on history of lobular carcinoma in situ (LCIS), atypia, family history of breast or ovarian cancer, genetic mutation, or history of chest wall radiation. As such, the population described are not easily comparable with the population in our clinic. It is interesting that only 2 women who chose to start chemoprevention in our cohort were at high-risk while uptake was greater in the moderate risk group (6% and 8% respectively). These women would not have been captured in a clinic which only included high-risk women.

For those of us who work in the field of breast cancer, even considering the potential for side effects, it is perplexing that women are not more willing to use medical options to reduce their risk of breast cancer. This is particularly the case as women could always stop the drug if they find the side effects are too intrusive. However, most of the women in this study were more concerned about avoiding additional medicines and side effects. While the option of taking low dose tamoxifen was discussed with women who raised concerns about side effects as their main barrier to considering preventative medicine [23] their reservations were more often about the concept than the numerical chance of side effects.

The conversation around preventative therapy is very different to that of a doctor treating cancer. Societally, we are not used to the idea of an ‘optional’ treatment. If preventative breast cancer medication could be marketed in a similar way to cardiovascular disease preventative medications, perhaps uptake would be much greater. Most patients who take statins or aspirin consider this to be quite ‘normal’. According to the British Heart Foundation, approximately 7–8 million people in the UK take statins [24]. If society’s view of breast cancer preventative therapy could be shifted in the same way, perhaps uptake would increase. This example is particularly striking given that the number needed to treat (NNT) for tamoxifen to prevent one breast cancer is 29 [25], while the NNT to prevent one atherosclerotic cardiovascular disease event using moderate intensity and high intensity statins was 30 and 20 respectively according to NICE definitions [26]. Tamoxifen has similar efficacy to statins, and yet statins are commonplace while tamoxifen as a preventative agent is still very unusual.

Although few women took up the offer of preventative therapy in the timescale of this study, they have been provided with the relevant information and some may choose to consider it again in the future. Raising awareness at the time of identification of risk may lead to more uptake over time. It is important that women know how to contact the clinical team when they feel timing is better for them.

While uptake in our clinic was low, preventative medicine may still be cost effective. With NNT of 29 for tamoxifen [25], and an average hourly rate for an advanced nurse practitioner in the UK of £25.73[27], we estimate it could cost approximately £9340 to prevent one breast cancer, assuming one hour of work per patient, and 363 prevention discussions to prevent one cancer. The cost of 1 year’s breast cancer treatment ranges from £5167 to £13,330 [28]. The specifics of healthcare financing in different countries warrant consideration in terms societal, provider and patient perspectives.

## Conclusion

Use of breast cancer risk calculators identifies more women at increased risk of breast cancer than prediction based on family history alone. We suggest that breast cancer risks calculations should be standard practice in symptomatic breast clinics. Those identified as “increased risk” should be offered enhanced screening and the opportunity to discuss preventative therapy. Reasons for not accepting medical therapy are diverse and important. The breast cancer community has yet to find the optimum timing and formula for discussing preventative therapy and must consider women’s own informed preferences above artificial targets.

## Data Availability

The datasets generated during and/or analysed during the current study are not publicly available because of ongoing analysis and interpretation, but data are available from the corresponding author on reasonable request.
